# A commentary on: “Anti-muscarinic adjunct therapy accelerates functional human oligodendrocyte repair”

**DOI:** 10.3389/fncel.2015.00274

**Published:** 2015-07-21

**Authors:** Fernando C. Ortiz, Maddalena Balia, David Orduz

**Affiliations:** Neurophysiology and New Microscopies Laboratory, INSERM U1128, Université Paris Descartes, Sorbonne Paris CitéParis, France

**Keywords:** muscarinic receptor, oligodendrocyte precursor cell (OPC), myelin repair, demyelinating diseases, *corpus callosum*

Unraveling the mechanisms that control the differentiation of oligodendrocyte precursor cells (OPCs) into mature myelinating oligodendrocytes (OLs) is central to improve the current treatments for demyelinating diseases such as multiple sclerosis (MS) (Franklin and ffrench-Constant, 2008). OPCs express functional receptors for glutamate, GABA, ATP, acetylcholine (AChR), and others (Butt et al., 2014; Hill and Nishiyama, 2014). Many studies have focused on the rodent OPC maturation, but less is known about human OPCs (hOPCs). Recently, Abiraman et al. (2015) demonstrated that treatment of forebrain hOPCs with a muscarinic AChR (mAChR) antagonist induces hOPCs differentiation into mature OLs *in vitro* and *in vivo*.

Abiraman et al. (2015) characterized human oligodendroglial cells by several techniques (i.e., FACS-based clustering, studies of phenotypic fate in cultured cells, immunohistochemical and mRNA expression profiles), defining three clear populations: (*i*) a progenitor population or authentic OPCs, (*ii*) a stable intermediate cell type between OPCs and OLs, a “pre-oligodendrocyte” population (preOL), and (*iii*) mature OLs. It is worth noting that in rodents, preOLs have been defined mostly by immunostaining profiles and it is not clear if they have a fleeting lifetime or they actually contribute as a stable subpopulation to the entire oligodendroglia lineage (Zuchero and Barres, 2013; Barateiro and Fernandes, 2014). Similar characterization should be conducted on rodent OPCs *in vivo*, to elucidate whether or not preOLs are a non-transient population in murines and also, until which extent rodent preOLs are comparable to their human counterparts.

Later, by using microarray analysis the authors found that M3 mAChR was a well-suited candidate to control hOPC differentiation into OLs. Application of muscarinic-agonist oxotremorine to hOPC cultures reduced the proportion of resultant OLs (Abiraman et al., 2015). Moreover, when co-cultures of hOPCs and human cholinergic neurons were treated with a selective M3 mAChR antagonist, the proportion of hOPCs-derived OLs increased. These results suggested that activation of M3 mAChRs inhibits OPC differentiation into OLs. To test this hypothesis *in vivo*, authors performed subcutaneous injections of solifenacin, a selective blood brain barrier-permeable M3 mAChR antagonist in both wild type mice and hypomyelinated transgenic mice shiverer/rag2, previously xenografted with hOPCs. They reported a premature induction of callosal OLs along with an increase in the myelin content, and a higher conduction velocity of xenografted fibers. Altogether these results suggest that (*i*) OPCs receive a tonic muscarinic input capable to inhibit their differentiation into mature OLs and that (*ii*) disruption of this muscarinic-dependent pathway can increase the number of differentiated OLs, which improve myelin repair.

A crucial unanswered point, however, is whether the muscarinic antagonist effect *in vivo* is mediated by direct action of mAChRs expressed by hOPCs. Experiments performed by subcutaneous solifenacin injections cannot entirely rule out the indirect activation of other cell types such as white matter astrocytes (Butt et al., 2014; Hill and Nishiyama, 2014) or interneurons (von Engelhardt et al., 2011) since the drug most likely reaches the entire brain. New directions must be defined to solve this fundamental question. If the reported effect is mediated by direct inhibition on M3 mAChR expressed in hOPCs, two main questions emerge. First, what is the endogenous source of acetylcholine in the *corpus callosum*? In the study reviewed here, experiments performed in wild type mice showed similar results to those using the xenografted hOPCs paradigm: an increased number of callosal OLs after solifenacin treatment. A similar result was reported in mice treated with benztropine, an anticholinergic M1/M3 mAChR agent (Deshmukh et al., 2013). Although enzymatic activity suggests cholinergic transmission in the pig white matter tracts (Hassel et al., 2008), to our knowledge there are no studies demonstrating acetylcholine release from human or rodent callosal fibers. Moreover, in rodents the great majority of axons constituting the *corpus callosum* are glutamatergic (Restani et al., 2009) and are capable of releasing glutamate in *bona fide* axon-OPC synapses in both control and demyelinating lesions (Ziskin et al., 2007; Sahel et al., 2015). In physiological conditions, a putative endogenous source of acetylcholine could be cortical and/or striatal axon terminals reaching the *corpus callosum* boundaries, as well as astrocytes present in white matter (Butt et al., 2014; Hill and Nishiyama, 2014). On the other hand, it is not known whether an inflammatory environment, such as in MS lesions, might favor a cholinergic transmission (Deshmukh et al., 2013). Further exploration is needed to determine these possibilities. The second question concerns the subcellular mechanism underlying the muscarinic-dependent effect. Stimulation of M3 mAChR initiates a signaling cascade resulting in the activation of the MAP kinase ERK1/2 pathway (Luo et al., 2008), which has been recently revealed as a key step in remyelination of the mouse *corpus callosum*, as it is involved in the correct transition from preOL to myelinating OLs (Michel et al., 2015). Thus, instead of improving myelin repair by promoting differentiation of OPCs into mature OLs, a disruption of a M3 muscarinic signaling could impair or delay the remyelination process, probably by inducing a cell arrest into preOL stage (Michel et al., 2015). This apparent paradoxical phenomenon deserves additional investigations.

In conclusion, Abiraman et al. (2015) shed light on the role of acetylcholine as a modulator of hOPCs differentiation, and more importantly, its potential implication on myelin repair. These results come when increasing interest on mAChRs emerges as a potential therapeutic target in MS, as proposed on rodent models (Deshmukh et al., 2013). How acetylcholine transmission occurs in human or rodent white matter, either in physiological or pathological conditions, remains an open question. Further studies are necessary to better characterize their cellular and subcellular mechanisms.

## Conflict of interest statement

The authors declare that the research was conducted in the absence of any commercial or financial relationships that could be construed as a potential conflict of interest.
